# Animal Histoplasmosis in Europe: Review of the Literature and Molecular Typing of the Etiological Agents

**DOI:** 10.3390/jof8080833

**Published:** 2022-08-09

**Authors:** Dunja Wilmes, Ursula Mayer, Peter Wohlsein, Michael Suntz, Jasmin Gerkrath, Christoph Schulze, Ina Holst, Wolf von Bomhard, Volker Rickerts

**Affiliations:** 1 Reference Laboratory for Cryptococcosis and Uncommon Invasive Fungal Infections, Division for Mycotic and Parasitic Agents and Mycobacteria, Robert Koch Institute, 13353 Berlin, Germany; 2 VetMed Labor GmbH–Division of IDEXX Laboratories, 70806 Kornwestheim, Germany; 3 Department of Pathology, University of Veterinary Medicine Hannover, 30559 Hanover, Germany; 4 State Institute for Chemical and Veterinary Analysis Freiburg, 79108 Freiburg, Germany; 5 Landeslabor Berlin-Brandenburg, Fb III-1 Pathologie, Bakteriologie, Fleischhygiene, 15236 Frankfurt (Oder), Germany; 6 Staatliches Tierärztliches Untersuchungsamt Aulendorf-Diagnostikzentrum, 88326 Aulendorf, Germany; 7 Fachpraxis für Tierpathologie, 80689 Munich, Germany

**Keywords:** histoplasmosis, *Histoplasma farciminosum*, animals, Europe, MLST, multilocus sequence typing, One Health, FFPE, ERG11, PRP, molecular epidemiology, cytochrome P450, human, animal, *Histoplasma capsulatum*, Germany

## Abstract

Histoplasmosis has been previously diagnosed in animals from Europe. The aim of this study is to review the literature on these reports, to analyze cases diagnosed at our laboratory (2000–2022) and to improve molecular typing of *Histoplasma capsulatum* directly from tissue to study the molecular epidemiology of *Histoplasma capsulatum* causing animal infections in Europe. Including 15 cases studied in our laboratory, we identified 39 cases of animal histoplasmosis between 1968 and 2022. They were diagnosed mostly in superficial tissue biopsies from cats and badgers from Central Europe. Using phylogenetic analyses of six partial genes, we were able to classify eight of the etiological agents as belonging to a highly supported lineage within the Eurasian clade. This study confirms the occurrence of autochthonous histoplasmosis in animals in Central Europe and proposes the addition of new loci to the MLST scheme to study the molecular epidemiology of histoplasmosis using either formalin-fixed paraffin-embedded tissue and fresh or cadaveric biopsies.

## 1. Introduction

Histoplasmosis is the most prevalent systemic infection caused by a thermal dimorphic fungal pathogen worldwide [1,2]. The main endemic areas include the Americas, Africa and Asia. In Europe, histoplasmosis in humans is generally considered to be imported, but some publications suggest autochthonous infections in Italy, United Kingdom, Germany, Turkey, Switzerland, Spain and France [3,4,5,6,7,8,9,10,11,12,13,14]. Several case reports and case series in wild and domestic animals from Europe seem to point in the same direction. Historically, the isolation of *Histoplasma capsulatum* (*H. capsulatum*) has been successful in the 1960s from the soil of a chicken yard near Bologna, Italy [15] and from bat feces in Romania [16]. However, since the onset of disease-associated symptoms in animals and in humans can occur years after travelling to highly endemic areas, it may be difficult to attribute the etiologic agent to a particular region. Regarding *H. capsulatum* in environmental samples, the fungus may be imported through organic products used as fertilizers [6,17] or by migrating defecating animals. Consequently, the geographical origin of the fungus or the infected subject are not reliable taxonomic classifiers. Instead, phylogenetic analysis should be aimed in this type of samples to connect the dots in order to better understand the molecular epidemiology of histoplasmosis in Europe.

The distinction of three *H. capsulatum* varieties is largely based on clinical manifestations and geographic origin. Besides *H. capsulatum* var. *capsulatum* and var. *duboisii*, which cause different types of histoplasmosis in humans, *H. capsulatum* var *farciminosum* (*H.c.f.*) causes epizootic lymphangitis in horses and other Equidae [18]. In 1999, a multilocus sequence typing (MLST) scheme for *H. capsulatum* isolates was developed [19,20]. Using the partial DNA sequences of four protein encoding genes (*arf*: ADP-ribosylation factor; *H-anti*: H antigen precursor; *ole1*: delta-9 fatty acid desaturase, *tub1*: alpha-tubulin), isolates can be attributed to at least eleven geographically delimited clades [20,21], which may differ by their virulence and drug susceptibility [22]. The authors reported that almost all equine *H. capsulatum* isolates, partially still defined as *H.c.f.* [18], clustered within the Eurasian clade [20,21], suggesting a susceptibility of horses to a particular type of *H. capsulatum*. However, these isolates do not form a highly supported monophyletic lineage [21]. As *H.c.f.* isolates clustered also with other clades (at least two: African and North American clade 2), the authors concluded that *H.c.f.* is not a valid taxon name.

In 2013, Arunmozhi Balajee et al. adapted the MLST scheme to type *H. capsulatum* from formalin-fixed paraffin-embedded (FFPE) tissue samples by designing primers to amplify shorter sequences of *arf*, *H-anti* and *tub1* [23]. Subsequently, histoplasmosis in a German badger [24] and in three German cats [25] could be attributed to the Eurasian clade by MLST. The usefulness of this approach is underscored by the difficulties of cultivation of *H. capsulatum* from animal samples due to frequent contamination of the agar plates by commensal microorganisms despite typical histopathological findings [23,26].

Here, we performed a review of the literature of histoplasmosis in European animals and described additional cases from *H. capsulatum*-infected animals, for which we participated in the diagnosis between 2000 and 2022. To test if *H. capsulatum* has a single genetic representative, we typed the etiological agents by MLST. In order to gain a higher resolution, three supplementary loci, including a part of the already-described PRP8 intein (*PRP8*) gene [27], and genes encoding for the cytochrome P450 enzyme lanosterol 14α-demethylase A (*CYP51pA*) and B (*CYP51pB*) were included for phylogenetic analysis. *PRP8* was chosen as it was proposed by Theodoro et al. [27] to identify cryptic species in the Latin American group A, from which the Eurasian clade emerges. *CYP51pA* and *CYP51pB* were chosen, since differences in antifungal susceptibility have been suspected in the phylogeographical clades.

## 2. Materials and Methods

### 2.1. Literature Review

We conducted a review of case reports, case series and other studies available in PubMed or Scopus according to PRISMA guidelines [28]. Electronic searches were performed in July 2022 with the following combinations of keywords: Europe AND (Histoplasma OR histoplasmosis) AND animals in SCOPUS. In PubMed, the search was performed by the combination of the following keywords: (Europe OR Albania OR Andorra OR Armenia OR Austria OR Azerbaijan OR Belarus OR Belgium OR Bosnia OR Herzegovina OR Bulgaria OR Croatia OR Cyprus OR Czech OR Denmark OR Estonia OR Finland OR France OR Georgia OR Germany OR Greece OR Hungary OR Iceland OR Ireland OR Italy OR Kazakhstan OR Kosovo OR Latvia OR Lichtenstein OR Lithuania OR Luxembourg OR Macedonia OR Malta OR Moldovia OR Montenegro OR Netherlands OR Norway OR Poland OR Portugal OR Romania OR Russia OR Serbia OR Slovakia OR Slovenia OR Spain OR Sweden OR Switzerland OR Turkey OR Ukraine OR United Kingdom) AND (Histoplasma OR histoplasmosis) AND animals and by the Medical Subject Headings (MeSH): (*“*Histoplasma*”*[Mesh] OR *“*Histoplasmosis*”*[Mesh]) AND *“*Europe*”*[Mesh] AND *“*Animals*”*[Mesh]. Additional records were identified by screening the referenced literature from these articles. Duplicates and cases published in more than one article were counted only once. The remaining articles were assessed for eligibility. The eligibility criteria were: case reports, case series and descriptive studies, proven histoplasmosis in animals in geographical Europe, with no reported travel history out of geographical Europe, and availability of an abstract or full text article.

The collected information included the tissue in which *H. capsulatum* could be detected, the species of the infected animal and diagnostic methods used to identify histoplasmosis. Additionally, the reported geographical origin of the animal was recorded. A map was made with qGIS3.14.1 for Desktop to visualize information on the inferred location of these infected animals as well as the affected species. Furthermore, the articles were screened for attribution of the etiological agent to a phylogenetic clade.

### 2.2. Tissue Specimens from Animal Histoplasmosis

Samples diagnosed as histoplasmosis that have been sent between 2000 and 2022 to our laboratory for diagnostic, and/or epidemiological studies of individual animals were retrieved from our database. Diagnostic criteria for histoplasmosis were in accordance with the revised definitions of the European Organization for Research and Treatment of Cancer (EORTC) and the Mycoses Study Group Education and Research Consortium (MSGERC) [29]. This entailed the isolation of *H. capsulatum*, histopathology or direct microscopy revealing the typical morphological features of histoplasmosis (2–4 µm narrow-based budding yeast cells) [29]. In the case of a histopathological diagnosis, histoplasmosis was confirmed by specific *H. capsulatum* PCRs or panfungal PCRs followed by amplicon identification by Sanger sequencing or hybridization on a chip [30,31]. Data on host, living environment, type of infection and diagnosis were provided by referring veterinaries.

### 2.3. Histoplasma Capsulatum Reference Isolates

Three *H. capsulatum* reference isolates from the Westerdijk Fungal Biodiversity Institute, formerly known as the CBS culture collection (Centraalbureau voor Schimmelcultures, now Westerdijk Fungal Biodiversity Institute, Utrecht, The Netherlands) were included in this study to obtain sequence information on the novel MLST loci. These were two *H. capsulatum* reference strains (CBS 477.64, CBS 478.64) originating from infections of horses in Poland (defined as *H.c.f* and already studied by Kasuga as H174 and H175 respectively [20]) and one *H. capsulatum* reference strain (CBS 136.72) from soil in the United States of America (Table S1). The reference isolates CBS 477.64 and CBS 478.64 were sent to the CBS in the year 1964 (communication from Ferry Hagen and Bert Gerrits van den Ende) and were isolated in Poland from enzootic outbreaks of lymphangitis epizootica equorum (personal communication from Dr S. Wołoszyn in a letter to Dr G.A. de Vries).

### 2.4. DNA Extraction

For DNA extraction of the fungal strains, we used a Master Pure^TM^ Yeast DNA purification kit (LGC Lucigen, Middleton, WI, USA) with an additional bead beating step using 0.25 mm silicon-carbide sharps (BioSpec Products Inc., Bartlesville, OK, USA) in a FastPrep-24^TM^5G machine (MP Biomedicals, LLC, Solon, OH, USA). Extracted DNA was eluted in 100 µL of TE buffer.

For fresh biopsies and for FFPE samples, the extraction of DNA was done until 2015 using the Maxwell^®^ 16 Tissue DNA Purification Kit as described in Bernhardt et al. [25]. For the FFPE samples arriving since 2015, the extraction was done using a Master Pure Yeast DNA Purification Kit (LGC Lucigen, Middleton, WI, USA) [32].

### 2.5. Molecular Tests

Typing fragments of the protein-encoding genes *arf*, *H-anti* and *tub1* (concatenated length*:* 1161 bp) were amplified from DNA extracted from cadaveric (2009-I, 2021-I), or FFPE tissue that arrived after 2018 (2018-I, 2018-II, 2020-I and 2021-II) as described by Kasuga [19]. For 2010-I and 2011-I, these sequences were already available as they were sequenced for a former publication [25]. The DNA from other samples obtained before 2018 (2014-I, 2015-I, 2010-II, 2012-I, 2014-II and 2017-I) was studied by the described MLST scheme [23]. In addition, we implemented *PRP8* [27], *CYP51A* [33] and *CYP51B* [34] loci, each in a long and in a short version. To design the new primers, an alignment ofsequences retrieved among Onygenales from FungiDB (http://FungiDB.org (accessed on 6 March 2022)) has been done. Primer sets were designed in conserved regions, including for *CYP51pA*, the region that harbors the point mutation Y136F, previously reported to occur in patients with a relapse during fluconazole therapy [34]. For PRP8, the regions showing the most differing single nucleotide polymorphisms between the available Eurasian isolates (H212, H205 and Tmu) were chosen. The DNAs arriving before 2018 were studied with the primers for the long version of these loci, and the other ones with the primers for the short version of these loci. The primer sequences, the amplicon lengths as well as the cycling conditions are given in Table S2.

The DNA of 2009–03 (a clinical *H. capsulatum* isolate) was used as a positive control and MilliQ water was used as a negative control. The PCR products were purified using an ExoSAP-IT^®^ kit (Thermo Fisher, Waltham, MA, USA) in a thermocycler for 15 min at 37 °C followed by 15 min at 80 °C. Forward and reverse strands of the PCR products were sequenced using the primers used for amplification in a Sanger Applied Biosystems 3500dx system (Life Technologies GmbH, Darmstadt, Germany). Forward and reverse sequences were assembled using Geneious Prime^®^ 2021.2.2 software (Biomatters, New Zealand).

All PCR reactions reported in this study were performed in a thermocycler (Biometra TAdvanced, AnalytikJena, Jena, Germany) using a final volume of 25 µL per PCR, including 24 µL of mastermix and 1 µL of extracted DNA. The mastermix consisted of PCR DreamTaq^TM^ Buffer 1× (Thermo Fisher Scientific, Schwerte, GE, Germany), MilliQ water, 0.2 mM of dNTP-mixed solution (Rapidozym, Berlin, GE, Germany), 1 pmol/mL of each primer and 0.05 U/mL of DreamTaq™ DNA-Polymerase (Thermo Fisher Scientific, Schwerte, GE, Germany). This resulted in additional sequence information of 2956bp (vs. 1161 bp Kasuga [19]) and 1019 bp (vs. 638 bp Arunmozhi Balajee [23]) for phylogenetic analysis.

### 2.6. Arf, H-anti, tub, PRP8, CYP51pA, CYP51pB in H. capulatum Whole Genome Sequences

Currently, there are six whole genome sequences of *H. capsulatum* isolates published (Table S1). For four of them (G217B, G186AR, H88 and H143), sequence information concerning *arf*, *H-anti* and *tub* was already published [20], and for H143*, PRP8* sequence information is also available [27] (Table S1). The search for the unpublished loci sequence information in the WGS of Nam1, G217B, G186AR, H88 and H143 was performed by a BLAST search of the corresponding amplicons in FungiDB (https://fungidb.org/, accessed on 6 March 2022) [35] and for Tmu, a *H. capsulatum* isolated in a patient from Taiwan, by BLASTing the corresponding amplicons in the downloaded WGS in Geneious Prime^®^ 2021.2.2 (Table S1).

### 2.7. Phylogenetic Analyses

#### 2.7.1. Arf-H-Anti-Tub

*Arf*, *H-anti* and *tub* sequences of *H. capsulatum* from an infected badger (named “Meles meles” in the trees and listed as #20 in Table 1) [24], of the herein described animal samples, of the WGS Nam1 and Tmu and of the reference isolates were included in this analysis. These sequences were all trimmed to the length of the amplicons described [19,23], excluding the primer binding sequences. Then, they were concatenated in the following order: *arf-H-anti-tub* ([20]: 1154 bp; [23]: 631 bp). These editing steps were performed in Geneious Prime^®^ 2021.2.2. The concatenated sequences were aligned by the MUSCLE [36] plugin 3.8.425 in Geneious Prime^®^ 2021.2.2 with the concatenated sequences of *H. capsulatum* isolates published by Kasuga et al. [20] and downloaded from the TreeBase database (http://www.treebase.org (accessed on 1 December 2021)) [20]These include four sequences from isolates H8, H82, H88, H143 for which WGS are available.

For the building of the phylogenetic trees, the Tamura-Nei genetic distance model, and as a resampling method, Neighbor Joining with 1000 bootstrap replications in Geneious Prime^®^ 2021.2.2 were used. Branch support was inferred by posterior probabilities by MrBayes [37], choosing as outgroup Nam1. Monophyletic groups that were supported by two methods (Bootstrap values ≥ 70 and posterior probabilities ≥ 0.95) were designated high confidence clades, as described by Teixeira et al. [21]. For visualization, the program iTOL 6.5.2 (iTOL: Interactive Tree Of Life (embl.de)) was used.

#### 2.7.2. Arf-H-Anti-Tub-PRP8-CYP51pA-Cyp51pB

Only the *Arf-H-anti-tub-PRP8-CYP51pA-Cyp51pB* sequences of the herein described animal samples, the reference isolates and the WGS were included. These were all trimmed as described above and then concatenated in the following order *arf-H-anti-tub-PRP8-CYP51pA-CYP51pB* (2956 bp). The concatenated sequences were aligned by the MUSCLE [36] plugin 3.8.425.

For the building of the phylogenetic trees, the same method as described above was used.

## 3. Results

### 3.1. Literature Review

**Figure 1:**
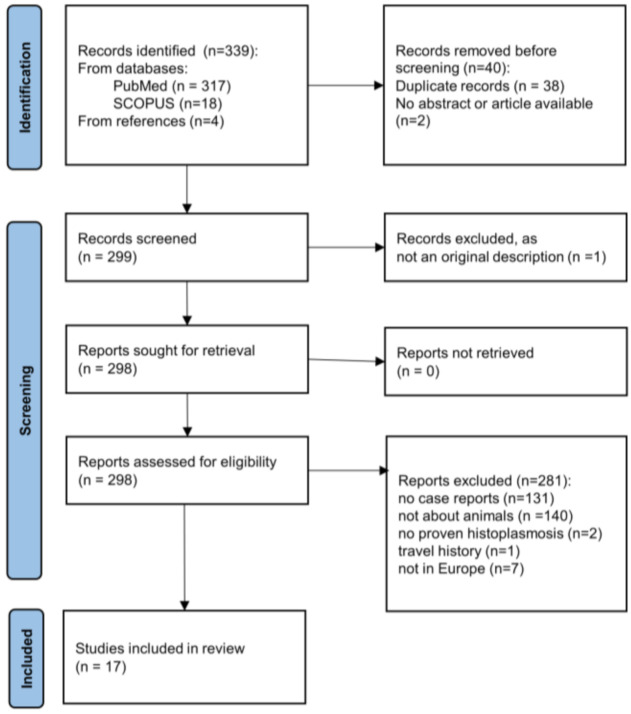
PRISMA flow diagram for the identification of publications considered for the inclusion into the review of animal histoplasmosis cases in Europe.

The literature search identified 335 articles [PubMed (*n* = 317) and SCOPUS (*n* = 18)] (Figure 1). Four additional articles [39,48,49,53] could be retrieved by screening for other histoplasmosis cases in the referenced literature of these identified articles [51,54]. Duplicate articles (*n* = 38) and article titles without an available abstract or article were excluded (*n* = 2). Another article [55] was excluded because of a replicate case report [39]. The 298 remaining articles were assessed by the eligibility criteria. Of these, 281 did not meet those: 131 did not report cases, 140 were about human cases, seven described cases in animals residing outside of geographical Europe, one was a cat with a travel history and two did not meet the criteria for proven histoplasmosis (Figure 1). A summary of the cases reported in the included studies is shown in Table 1.

**Figure 2:**
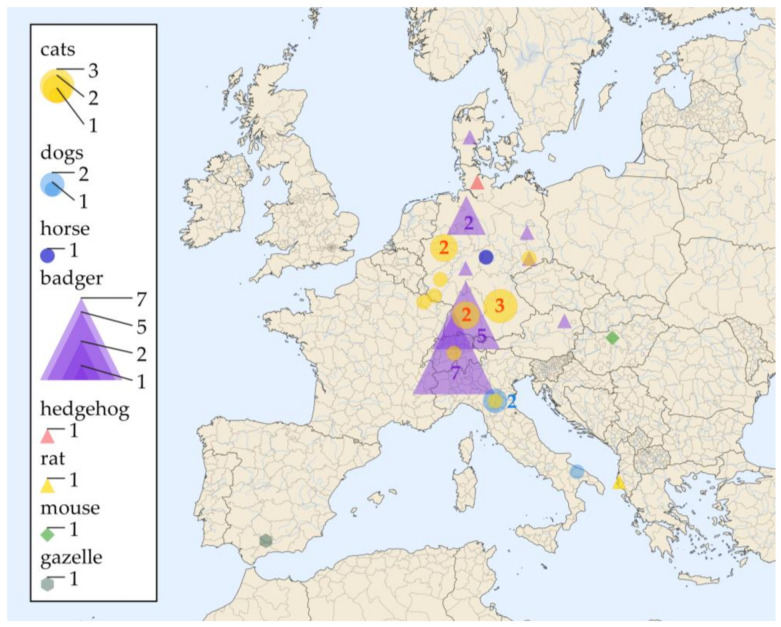
Living environment of 39 animal cases of proven histoplasmosis reported in Europe between 1968 and 2022. In Germany, the cases were distributed over the whole country. Sizes of the symbols represent the numbers of cases. If more than one case was detected, the numbers are indicated near the symbol in the corresponding color.

In total, including the newly described cases in this article, there have been reports on 39 cases in wild animals (European badgers (*Meles meles*): *n* = 19, European hedgehog (*Erinaceus europaeus*): *n* = 1; black rat (*Rattus rattus*): *n* = 1), domesticated animals (cats (*Felis catus*): *n* = 12, dogs (*Canis lupus familiaris*): *n* = 3, horse (*Equus caballus*): *n* = 1) a captive exotic animal (gazelle (*Gazella dorcas neglecta*) and one laboratory mouse (*Mus musculus*) through Europe (Figure 2, Table 1).

Most of the cases were reported from Germany (*n* = 22; 56.4%), followed by Switzerland (*n* = 8; 20.5%) and Italy (*n* = 3; 7.7%). In Albania, France, Denmark, Austria, Hungary and Spain, one animal per country was reported with histoplasmosis (2.6% each) (Figure 2).

Most cases were diagnosed based on samples from skin lesions (*n* = 29; 74%), but there were also cases for which no skin lesions were reported (*n* = 10; 26%) (Table 1). In five cases, a lung infection could be proven (13%), and in two cases, *H. capsulatum* could be isolated from peribronchial lymph nodes (5%) [51] (Table 1).

Diagnosis was based mainly on histopathological studies (36 cases; 92%) confirmed by PCR, immunohistology or a combination of both, while three cases were diagnosed by culture (8%) (Table 1; #31, #32 and #39).

### 3.2. Tissue Specimens from Veterinary Histoplasmosis Cases

Fifteen cases of histoplasmosis in animals between 1 January 2000 and 31 December 2021 at our Institute fulfilled the inclusion criteria. The majority of them were cats (*n* = 10; 66.6%) together with five badgers (33.3%). The diagnosis was based on histopathology in all of them and confirmed by different PCR assays (Table 1). Most of the samples were FFPE samples, and only two were cadaveric biopsies from badgers (Table S3). In two cases, *H. capsulatum* could also be retrieved from tissues other than skin (# 5, # 7 in Table 1). Four of them have already been published [25,45] (#2, #3 and #15 in Table 1). Of these animals, 14 were from Germany (Baden Wuerttemberg *n* = 4, Bavaria *n* = 3, North Rhine-Westphalia *n* = 2; Saarland *n* = 1, Brandenburg *n* = 1, Lower Saxony *n* = 1, Rhineland-Palatinate: *n* = 1; Saxony: *n* = 1) and one was from France (Figure 2). For one of those samples (#15 in Table 1), no DNA was left for further studies.

### 3.3. Results of the Multilocus Sequence Typing

The success rate of amplification by PCRs on the reference isolates and on the veterinary samples are given in Table S3. Briefly, for all reference isolates as well as for five of the etiological agents of animal histoplasmosis for which DNA was still available (*n* = 14), fragments of all six gene loci could be amplified and sequenced in their long version. For five (21.4%) animal samples, all six gene loci could be amplified and sequenced in their short version (Table S3). The corresponding accession numbers can be found in Table S1.

### 3.4. Phylogenetic Analyses

#### 3.4.1. Arf-H-Anti-Tub

**Figure 3:**
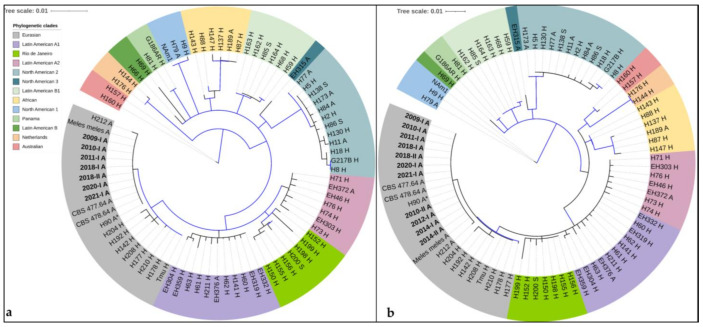
Phylogenetic analyses by neighbor-joining of *H. capsulatum* from the herein analyzed tissue samples of animals (in bold letters) using published three gene MLST schemes (*arf-H-anti-tub)* for isolates (1154 bp) [20] (**a**) and for pathology blocks (631 bp) [23] (**b**). As reference, a published alignment was used [20]. Both schemes show clustering of the herein described animal samples with the Eurasian clade. * H90 has the identical multilocus genotype as 10 other *H. capsulatum* var. *farciminosum* isolates; _A: of animal origin; _H: of human origin; _S: from soil. Clades were named as described by Teixeira et al. [21] and Vite-Garín et al. [56] and are represented by different colors as described in the legend. Bootstraps above 70% are indicated by a blue line.

Seven animal samples (2009-I, 2010-I, 2011-I, 2018-I, 2018-II, 2020-I and 2021-I) for which the three partial genes (*arf*, *H-anti* and *tub*) as described by Kasuga et al. [19] could be amplified and sequenced (Table S3), clustered with the Eurasian clade with a bootstrap support of 98.3% and a posterior probability of 1 (Figure 3a). Within the Eurasian clade, they clustered with eleven *H.c.f* isolates [20], the sequences from a histoplasmosis case in a German badger (*Meles meles*, #20 in Table 1) [24] and two reference isolates from horses in Poland (CBS 477.64 or H174 and CBS 478.64 or H175) with a bootstrap support of 61.4% and a posterior probability of 0.94. This did not meet the criteria for a highly supported monophyletic group (Figure 3a).

For four supplementary animal samples (2010-II, 2012-I, 2014-I and 2014-II), the partial genes as described by Arunmozhi Balajee [23] could be amplified (Table S3). In the phylogenetic analysis of these sequences with those from above, all animal samples clustered in the Eurasian clade with a bootstrap support of 77.1% and a posterior probability of 1 (Figure 3b). Additionally, the animal samples clustered within the Eurasian clade with eleven *H.c.f.* isolates, the sequences from *Meles meles* [24] and two reference isolates from horses in Poland with a bootstrap support of 61.4% and a posterior probability of 0.93, which did not meet the criteria for a monophyletic group (Figure 3b).

#### 3.4.2. Arf-H-Anti-Tub-PRP8-CYP51pA-Cyp51pB

**Figure 4:**
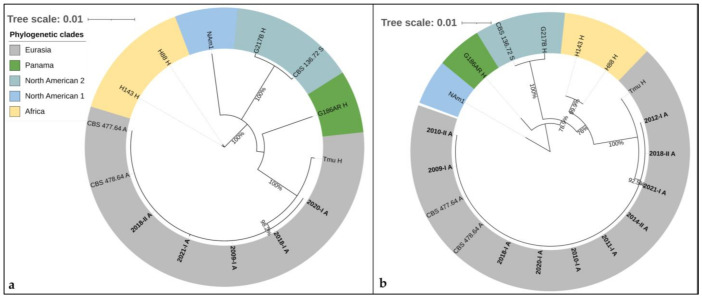
Phylogenetic analyses by neighbor-joining of *H. capsulatum* from animal tissue samples and isolates by MLST schemes (*Arf-H-anti-tub-PRP8-CYP51pA-Cyp51pB)* with three supplementary partial genes (2956 bp) (**a**) and with reduced allele size for amplification from FFPE blocks (1019 bp) (**b**). The addition of this genetic information may increase the resolution of the Eurasian clade. The herein studied *H. capsulatum* samples from animals (in bold letters) clustered together in a distinct highly supported group, clearly distinguishable from the other Eurasian isolate (Tmu). _A: of animal origin; _H: of human origin; _S: from soil. Clades were named as described by Teixeira et al. [21].

The phylogenetic analysis of these loci was performed on the concatenated sequences of *arf-H-anti-tub-PRP8-CYP51pA-Cyp51pB* of five veterinary samples (2009-I, 2018-I, 2018-II, 2020-I and 2021-I) (Table S3), all reference isolates and the sequences of the WGSs in the long version (2956 bp). All veterinary samples clustered within the Eurasian clade (bootstrap value 100%, posterior probability 1) with two CBS reference isolates of *H.c.f.* (CBS 477.64 and CBS 478.64) in a highly supported lineage (bootstrap value 98.2%, posterior probability 1), clearly distinguishable from Tmu (Figure 4a).

For five additional animal samples (2010-I, 2010-II, 2011-I, 2012-I and 2014-II), all short sequences could be amplified, concatenated and aligned (1019 bp) (Table S3). They all clustered together with the aforementioned animal samples (2009-I, 2018-I, 2018-II, 2020-I and 2021-I) within the Eurasian clade (bootstrap support 100%, posterior probability 1) in a distinct lineage with a bootstrap support of 92.5% and a posterior probability of 1 (Figure 4b).

## 4. Discussion

Our article reviews previous reports on histoplasmosis in animals in Europe and adds new cases diagnosed at our laboratory. In addition, we tried to improve the methods to type *H. capsulatum* directly from tissue samples of infected animals by increasing the size of the analyzed concatenated sequences, extending from 1154 to 2956 bp and from 631 to 1019 bp. We thus confirmed that these infections were caused by closely related fungi, potentially representing a subclade within the Eurasian clade. This could be due to a clonal evolution of *H. capsulatum* from one or a few South American strains that were transported to Europe in the late 1400s and early 1500 [57]. A similar phenomenon has previously been described for *H. capsulatum* infections in cats clustering in a subclade of Nam1 [23], and for *H. capsulatum* isolated from bats clustering in a clade (Nam3) closely related to Nam2 [56]. However, as the number of included sequences was limited, further studies would be necessary to assess the discriminatory power of these new loci and to evaluate the benefit of including them into the MLST schemes.

Most of the cases were described in Central Europe (Table 1 and Figure 2), but since the data was extracted mostly from case reports, we do not know the true prevalence of histoplasmosis among animals in Europe. The preponderance of cases reported from Germany may be a simple publication bias, since specific *Histoplasma* PCRs have been widely available in Germany for a long time [58,59]. While the infections in domesticated animals, a captive exotic animal and a laboratory mouse may always be suspected to be imported, the fact that 21 of the cases were diagnosed in local wildlife is an important argument for the presence of *H. capsulatum* in the living environment of these animals. A potential limitation of this literature review is that two articles had to be excluded as we could not find the articles nor the abstracts. Furthermore, another limitation is linked to the sources of PubMed and SCOPUS. Articles which are not included in MEDLINE and EMBASE may have been missed.

Overall, most of the reported animals showed cutaneous lesions (*n* = 28; 72%) and one horse showed keratitis. These are also typical presentations of epizootic lymphangitis caused by *H.c.f.* which include cutaneous pyogranulomas with lymphangitis, conjunctivitis or multifocal pneumonia [60,61]. Contrary to histoplasmosis in humans, isolated pulmonary involvement was not described for cases detected in Central Europe, and disseminated disease seems to be rare. This leads to the hypothesis that the mode of infection in animals in Central Europe could be by direct contact, as already suggested for *H.c.f.* [18,60]. Interestingly, the clinical presentation of histoplasmosis in animals from Italy, Spain, Albania and Hungary (*n* = 5) seems to be different, as in all these animals, no cutaneous lesions were reported, and in three, the dissemination of the etiological agent could be proven. Since the number of reported cases is limited, this observation may be due to undersampling or it may be a hint for the presence of a *H. capsulatum* variety with altered properties in these regions. Overlapping geographical distributions of different varieties and clades of *H. capsulatum* have been described in multiple regions [2,21], but phylogenetic analyses are missing to prove it in Europe.

Most of the diagnosis in wild animals is made at necropsy, which may render cultural recovery of *H. capsulatum* difficult due to the colonization of the carcasses by fast-growing bacteria and fungi during post-mortem heterolysis [24]. Increasing the awareness of veterinarians may be a way of ascertaining access to fresh biopsies from cases in domestic or exotic animals for further processing. It should be adapted to the needs using fungi-specific media and appropriate extended incubation times. However, a culture may still fail, since adapted culturing of fresh biopsies has been tried with samples from six infected dogs in Japan without success [26]. Failed cultures may further be related to extremely slow growth of the agent as observed for *H.c.f.* [18]. We did not find any case report of animal histoplasmosis in Europe suspected by a positive antibody or antigen detection, probably because most of the cases were diagnosed due to suggestive histopathological images and not by the clinical presentation.

Since the 1950s, there have been repeatedly reported cases of autochthonous human histoplasmosis in Europe [4,6,7,8,10,12,13,14,62,63]. However, as the period between travel to highly endemic areas and onset of clinical symptoms may vary considerably, truly autochthonous etiology likely can only be proven by phylogenetic analysis. Thus, this should be aimed in samples from humans, animals and the environment to elucidate the epidemiology of histoplasmosis in Europe by molecular techniques. The relationship of Eurasian *H. capsulatum* strains in animals with the strains causing autochthonous disease in humans in Europe remains unclear, since for the latter, no phylogenetic analysis has ever been described. Molecular characterization of this fungus in animals in Europe relies mainly on biopsies. The molecular characterization by MLST [20,23] confirmed for the eleven herein studied *H. capsulatum* of animal samples the clustering within the Eurasian clade, especially with eleven of thirteen *H.c.f.* isolates. By the addition of supplementary gene fragments, the resolution of the phylogenetic analysis may be increased. It confirms the monophyletic nature of the group within the Eurasian clade, clearly differing from the MLST sequences of Tmu, a Eurasian isolate that infected a human in Taiwan. These findings are a first step towards a higher resolution of *H. capsulatum* strains belonging to the Eurasian clade and the elucidation of *H. capsulatum* epidemiology in Europe. This knowledge can be used as a tool to study isolates and biopsy blocks from suspected autochthonous infections in Europe to get a deeper understanding on the epidemiology of the disease.

Besides the supplementary phylogenetic information, the sequencing of *CYP51pA* may be used to study the frequency of the point mutation Y136F, which has been described in *H. capsulatum* isolates with decreased sensitivity to fluconazole and voriconazole [34]. This point mutation aligns with Y132 in *Candida* and Y121 in *Aspergillus* species [64]. In Germany, Y121F mutations have been found among others in six environmental azole-resistant *Aspergillus fumigatus* isolates [64]. The analysis of these sequences may add valuable information of the possible resistance pattern of *H. capsulatum*, even if larger studies describing this mechanism are lacking. In the current study, no such point mutation was identified.

In the meantime, health surveillance on domesticated and wild animals, including bats and badgers, would be helpful to getting a realistic idea of the prevalence and geographical distribution of histoplasmosis in European animals and to detecting microfoci and defining the niche of *H. capsulatum* in the European environment. The best method for the health surveillance of *H. capsulatum* in animals remains an open question. Seroepidemiological studies have been done in dogs with different methods [65,66], however, the sensitivity and specificity of serologic tests in different animal populations is poorly studied and may be decreased in animals without disseminated disease [67]. The detection of such environmental foci might shed light onto the suspected autochthonous human cases and help target the efforts to culture *H. capsulatum* belonging to this lineage for more in-depth molecular and virulence studies.

## Figures and Tables

**Table 1:** Animal cases of putative autochthonous histoplasmosis diagnosed or reported from Europe since 1968.

Nr	Identificator	Living Environment: Country (State)	Animal	Positively Sampled Tissues	Diagnosis	Supplementary Diagnostic Methods	Reference
1	2009-I	Germany (BB)	badger	skin	histopathology	PCR (18SrDNA ^2^, ITS2 rDNA ^3^)	this study
2	2010-I	Germany (BY)	cat	skin	histopathology	PCR (100 kDa-like protein ^6^, ITS2 rDNA ^3^), MLST (Eurasian clade)	[25]
3	2011-I	Germany (RP)	cat	skin	histopathology	PCR (100 kDa-like protein ^6^, ITS2 rDNA ^3^), MLST (Eurasian clade)	[25]
4	2012-I	France (Lorraine)	cat	skin	histopathology	PCR (18SrDNA ^2^, ITS2 rDNA ^3^)	this study
5	2014-I	Germany (BW)	badger	skin, testicles	histopathology	PCR (18SrDNA ^2^, ITS2 rDNA ^3^)	this study
6	2015-I	Germany (SN)	badger	skin	histopathology	PCR (18SrDNA ^2^, ITS2 rDNA ^3^)	this study
7	2021-I	Germany (BW)	badger	skin, regional lymph node	histopathology	PCR (*H* qPCR ^4^)	this study
8	2010-II	Germany (SL)	cat	skin	histopathology	PCR (18SrDNA ^2^, ITS2 rDNA ^3^)	this study
9	2014-II	Germany (BY)	cat	skin	histopathology	PCR (28SrDNA qPCR ^5^)	this study
10	2017-I	Germany (BW)	cat	skin	histopathology	PCR (*H* qPCR ^4^)	this study
11	2018-I	Germany (BW)	cat	skin	histopathology	PCR (*H* qPCR ^4^)	this study
12	2018-II	Germany (NW)	cat	skin	histopathology	PCR (*H* qPCR ^4^)	this study
13	2020-I	Germany (NW)	cat	skin	histopathology	PCR (*H* qPCR ^4^)	this study
14	2021-II	Germany (NI)	badger (A)	skin, regional lymph nodes, spleen, testicle	histopathology	immunohistology	
15	n.a.	Germany (BY)	cat	skin	histopathology	PCR (100 kDa-like protein^6^, ITS2 rDNA ^3^), MLST (Eurasian clade)	[25]
16	n.a.	Germany (BW)	badger	skin	histopathology	immunohistology	[38]
17	n.a.	Germany (BW)	badger	skin	histopathology	immunohistology	[38]
18	n.a.	Germany (BW)	badger	skin	histopathology	immunohistology	[38]
19	n.a.	Germany (NI)	badger (B)	skin, regional lymph nodes, spleen	histopathology	immunohistology	[39]
20	Meles meles	Germany (HE)	badger	skin and regional lymphnode	histopathology	PCR (ITS1-5.8S-ITS2 ^7^), MLST (Eurasian clade)	[24]
21	n.a.	Germany (SH)	hedgehog	spleen, liver, lung, bone marrow, lymph nodes, myocardium, kidney	histopathology	PCR (100 kDa-like protein ^6^, 18SrDNA ^2^)	[40]
22	n.a.	Germany (unknown)	horse	cornea	histopathology	n.a.	[41]
23	n.a.	Switzerland (Bern)	badger	submandibular lymph node	histopathology	immunohistology	[42]
24	n.a.	Switzerland	badger	skin	histopathology	n.a.	[43]
25	n.a.	Switzerland	badger	skin	histopathology	n.a.	[43]
26	n.a.	Switzerland	badger	skin	histopathology	n.a.	[43]
27	n.a.	Switzerland	badger	skin	histopathology	n.a.	[43]
28	n.a.	Switzerland	badger	skin, subcutaneous lymph nodes, lungs	histopathology	n.a.	[43]
29	n.a.	Switzerland	badger	skin, subcutaneous lymph nodes, lungs	histopathology	n.a.	[43]
30	n.a.	Switzerland	cat	skin	histopathology	PCR (28S rDNA ^8^)	[44]
31	n.a.	Italy (ER)	dog	peribronchial lymph nodes	culture	n.a.	[45]
32	n.a.	Italy (ER)	dog	peribronchial lymph nodes	culture	n.a.	[45]
33	n.a.	Italy (Apulia)	dog	epidural spinal cord	histopathology	immunohistology, PCR (ITS1-5.8S-ITS2 ^8^)	[46]
34	n.a.	Italy (ER)	cat	lung, abdominal mass	histopathology	immunohistology	[47]
35	n.a.	Albania (Vlorë)	rat	spleen, liver	histopathology	n.a.	[48]
36	n.a.	Hungary	laboratory mouse	liver, peritoneal liquid	histopathology	n.a.	[49]
37	n.a.	Denmark (NJ)	badger	skin, liver, kidney, lymph node	histopathology	immunohistology	[50]
38	n.a.	Austria (NOE)	badger	skin, regional lymph nodes	histopathology	immunohistology	[51]
39	n.a.	Spain (Andalusia)	Gazelle ^1^	lung, intestines, spleen, kidneys, myocardium, liver	culture, histopathology	n.a.	[52]

BB: Brandenburg; BW: Baden Wuerttemberg; BY: Bavaria; ER: Emilia-Romagna; HE: Hessen; NI: Lower Saxony; NJ: North Jutland; NOE: Lower Austria; NW: North Rhine-Westphalia; RP: Rhineland-Palatinate; SH: Schleswig-Holstein; SN: Saxony; SL: Saare; n.a.: not applicable or unspecified, MLST: multilocus sequence typing; ^1^ originating in a captive breeding center in Spain; ^2^ nested PCR targeting the 18S rDNA; ^3^ PCR targeting a part of the ITS2 region with amplicon identification by hybridization; ^4^ Histoplasma-specific Taqman qPCR detecting a region of the ITS1 rDNA; ^5^ qPCR targeting the 28S rDNA region; ^6^ nested PCR targeting the Histoplasma-specific 100 kDa protein; ^7^ conventional PCR targeting the ITS1-ITS2 region (primer ITS-1 and ITS4) with amplicon identification by sequencing; ^8^ conventional PCR targeting the 28S rDNA region with amplicon identification by sequencing.

## Data Availability

Not applicable.

## Supplementary Materials

The following supporting information can be downloaded at: https://www.mdpi.com/article/10.3390/jof8080833/s1, Table S1: Accession numbers of gene loci amplified from tissue samples of animals with histoplasmosis and of reference isolates reported in this study as well as the localization of them within the published whole genome sequences; Table S2: Herein described new primers and PCR conditions; Table S3: Results of the PCRs as well as of the sequencing of the different gene loci in the different studied materials containing *H. capsulatum.*
