# Supplementary material for: Animal Histoplasmosis in Europe: Review of the Literature and Molecular Typing of the Etiological Agents
Source: J Fungi (Basel). 2022 Aug 9;8(8):833. doi: 10.3390/jof8080833 (PMC9410202; doi:10.3390/jof8080833)
Supplement: Supplementary file 1 [file jof-08-00833-s001.zip › Table S1_Accession numbers.pdf]

Table S1: Accession numbers of gene loci amplified from tissue samples of animal with histoplasmosis and of reference isolates reported in this study as well as the localization of them within the published whole genome sequences

Identifier	other names	origin	*arf*	*H-anti*	*tub*	*PRP8*	*cyp51pA*	*cyp51pB*
CBS 136.72	n.a.	soil (USA)	OM837779	OM816641	OM869470	OM731540	OM816659	OM675767
CBS 477.64	H174	horse (Poland)	OM837785	OM816648	OM869477	OM731547	OM816666	OM675774
CBS 478.64	H175	horse (Poland)	OM837786	OM816649	OM869478	OM731548	OM816667	OM675775
Tmu	n.a.	human	AMXR01000497.1 (6836-7316) contig_511	AMXR01007615.1 (17 738- 18 172) contig_7912	AMXR01008776.1 (15 914-16 219) contig_9148	AMXR01000209.1 (11 645-11 077) contig_218	AMXR01010037.1 (12 362-13 007) contig_10501	AMXR01008275.1 (18 251-18 859) contig_8641
H88	ATCC32281/ 2438/RV26821	human	Kasuga, 2003	Kasuga, 2003	Kasuga, 2003	DS990639 (2 658 737- 2 659 296)	DS990645 (403 243- 403 888)	DS990639 (2 387 790-2 388385)
H143	4719/CBS 287.54	human	Kasuga, 2003	Kasuga, 2003	Kasuga, 2003	JX274641	GG692438 (397 218- 397 803)	GG692421 (1 851 111- 1 851 706)
G186AR	H82	human	Kasuga, 2003	Kasuga, 2003	Kasuga, 2003	GCA_017355575.1 (1 557 354-1 557 910)	GG663383 (81 075- 81714)	GG663363 (1 288 426-1 289 021)
G217B	H8/ATCC26032/ 1001/IFM41659	human	Kasuga, 2003	Kasuga, 2003	Kasuga, 2003	HISTO_FE.Contig19 (886 708-887 264)	HISTO_GB.Contig114 (91 444-92 086)	HISTO_ZL.Contig1161 (480 730-481 325)
Nam1	n.a.	unknown	CH476655 (648 6055-648 6516)	CH476655 (626 230- 626 638)	CH476655	CH476658 (1 871 717- 1 872 276)	CH476658 (84 405- 85 050)	CH476658 (1 586 773- 1 587 367)
2009-I	n.a.	badger	OM837780	OM816642	OM869471	OM731541	OM816660	OM675758
2014-I	n.a.	badger	OM837774	OM816638	OM869465	OM731535	OM816653	n.a.
2015-I	n.a.	badger	OM837776	n.a.	OM869467	n.a.	OM816655	n.a.
2021-I	n.a.	badger	OM914585	OM816640	OM869469	OM731539	OM816658	OM675766
2010-II	n.a.	cat	OM837773	OM816636	OM869463	OM731533	OM816651	OM675761
2014-II	n.a.	cat	OM837775	OM816639	OM869466	OM731536	OM816654	OM675763
2017-I	n.a.	cat	OM837777	n.a.	n.a.	OM731537	OM816656	OM675764
2018-I	n.a.	cat	OM837783	OM816645	OM869474	OM731544	OM816663	OM675771
2018-II	n.a.	cat	OM914586	OM816646	OM869475	OM731545	OM816664	OM675772

2020-I	n.a.	cat	OM837784	OM816647	OM869476	OM731546	OM816665	OM675773
2012-I	n.a.	cat	OM914584	OM816637	OM869464	OM731534	OM816652	OM675762
2010-I	n.a.	cat	OM837781	OM816643	OM869472	OM731542	OM816661	OM675769
2011-I	n.a.	cat	OM837782	OM816644	OM869473	OM731543	OM816662	OM675770
2021-II	n.a.	badger	OM837778	n.a.	OM869468	OM731538	OM816657	OM675765

n.a.: not applicable
