# Supplementary material for: Animal Histoplasmosis in Europe: Review of the Literature and Molecular Typing of the Etiological Agents
Source: J Fungi (Basel). 2022 Aug 9;8(8):833. doi: 10.3390/jof8080833 (PMC9410202; doi:10.3390/jof8080833)
Supplement: Supplementary file 1 [file jof-08-00833-s001.zip › Table S2 Herein described new primers and PCR conditions.pdf]

Table S2: Herein described new primers and PCR conditions

Gene locus	Primer name: sequence[a]	Amplicon size (bp)	PCR conditions
PRP8	fw: CTTCAGTCGTTTCCCCTG<br>rev: TGCTGCTCTGTAGAAACACT	560	Initial denaturation step (3min) at 95°C<br>DNA denaturation step (15s) at 94°C<br>Annealing step (30s) at 65°C (-0.7°C/cycle) for the next 12 cycles, thereafter at 56°C for 20 cycles
	fw FFPE: GAAGCCGATGAAATCCAGGG<br>rev FFPE: TATCAGGAGTGCCAACAGGT	111	Extension step (60s) at 72°C for 35 cycles (45 cycles for FFPE and fresh biopsy)<br>Final extension step (5min) at 72°C
CYP51pA	fw: AGACTACCGTGTTTCTTGGAA<br>rev: GCAATCTCGATGTCTGGGA	646	Initial denaturation step (3min) at 95°C<br>DNA denaturation step (15s) at 95°C<br>Annealing step (30s) at 55°C
	rev FFPE: TCCATCAGCTTCGAGTTTGG	110	Extension step (60s) at 72°C for 35 cycles (45 cycles for FFPE and fresh biopsy)<br>Final extension step (5min) at 72°C
CYP51pB	fw: GCACGGCGACATCTTCAC<br>rev: TACTTGATGAACTTCTTCTGCT	596	Initial denaturation step (3min) at 95°C<br>DNA denaturation step (15s) at 95°C<br>Annealing step (30s) at 55°C
	rev FFPE: TACTTGATGAACTTCTTCTGCT	170	Extension step (60s) at 72°C for 35 cycles (45 cycles for FFPE and fresh biopsy)<br>Final extension step (5min) at 72°C

[a] All primer sequences are in 5′ to 3′ configuration. fw: forward; rev: reverse; *PRP8*: PRP8 intein; *CYP51pA*: cytochrome P450 enzyme lanosterol 14α-demethylase A; *CYP51pB*: Cytochrome P450 enzyme lanosterol 14α-demethylase B; FFPE: formalin-fixed paraffin-embedded samples)
