# Supplementary material for: Animal Histoplasmosis in Europe: Review of the Literature and Molecular Typing of the Etiological Agents
Source: J Fungi (Basel). 2022 Aug 9;8(8):833. doi: 10.3390/jof8080833 (PMC9410202; doi:10.3390/jof8080833)
Supplement: Supplementary file 1 [file jof-08-00833-s001.zip › Table S3_Results of the PCRs.pdf]

Table S3: Results of the PCRs as well as of the sequencing of the different loci in the different studied materials containing *H. capsulatum*

sample identifier	clade[a]	sample type	*arf*		*H-anti*		*tub1*		*PRP8*		*CYP51pA*		*CYP51pB*	
			long*	short**	long*	short**	long*	short**	long	short	long	short	long	short
2009-I	Eurasia (new)	cadaveric	pos.	n.a.	pos.	n.a.	pos.	n.a.	pos.	n.a.	pos.	n.a.	pos.	n.a.
2010-I	Eurasia (new)	FFPE	pos.	n.a.	pos.	n.a.	pos.	n.a.	n.a.	pos.	n.a.	pos.	n.a.	pos.
2010-II	Eurasia (new)	FFPE	n.a.	pos.	n.a.	pos.	n.a.	pos.	n.a.	pos.	n.a.	pos.	n.a.	pos.
2011-I	Eurasia (new)	FFPE	pos.	n.a.	pos.	n.a.	pos.	n.a.	n.a.	pos.	n.a.	pos.	n.a.	pos.
2012-I	Eurasia (new)	FFPE	n.a.	pos.	n.a.	pos.	n.a.	pos.	n.a.	pos.	n.a.	pos.	n.a.	pos.
2014-I	Eurasia (new)	FFPE	n.a.	pos.	n.a.	pos.	n.a.	pos.	n.a.	pos.	n.a.	pos.	n.a.	sf
2014-II	Eurasia (new)	FFPE	n.a.	pos.	n.a.	pos.	n.a.	pos.	n.a.	pos.	n.a.	pos.	n.a.	pos.
2015-I	Eurasia	FFPE	n.a.	pos.	n.a.	neg.	n.a.	pos.	n.a.	pos.	n.a.	sf	n.a.	sf
2017-I	Eurasia	FFPE	n.a.	pos.	n.a.	neg.	n.a.	neg.	n.a.	pos.	n.a.	pos.	n.a.	pos.
2018-I	Eurasia (new)	FFPE	pos.	n.a.	pos.	n.a.	pos.	n.a.	pos.	n.a.	pos.	n.a.	pos.	n.a.
2018-II	Eurasia (new)	FFPE	pos.	n.a.	pos.	n.a.	pos.	n.a.	pos.	n.a.	pos.	n.a.	pos.	n.a.
2020-I	Eurasia (new)	FFPE	pos.	n.a.	pos.	n.a.	pos.	n.a.	pos.	n.a.	pos.	n.a.	pos.	n.a.
2021-I	Eurasia (new)	cadaveric	pos.	n.a.	pos.	n.a.	pos.	n.a.	pos.	n.a.	pos.	n.a.	pos.	n.a.
2021-II	Eurasia	FFPE	neg.	pos.	neg.	neg.	neg.	pos.	neg.	pos.	neg.	pos.	neg.	pos.
CBS 136.72	Nam2	isolate	pos.	n.a.	pos.	n.a.	pos.	n.a.	pos.	n.a.	pos.	n.a.	pos.	n.a.
CBS 477.64	Eurasia (new)	isolate	pos.	n.a.	pos.	n.a.	pos.	n.a.	pos.	n.a.	pos.	n.a.	pos.	n.a.
CBS 478.64	Eurasia (new)	isolate	pos.	n.a.	pos.	n.a.	pos.	n.a.	pos.	n.a.	pos.	n.a.	pos.	n.a.

arf: ADP-ribosylation factor; *H-anti*: H antigen precursor; *tub1*: alpha-tubulin; *PRP8*: PRP8 intein; *CYP51pA*: cytochrome P450 enzyme lanosterol 14α-demethylase A; *CYP51pB*: Cytochrome P450 enzyme lanosterol 14α-demethylase B; *as described by Kasuga; **as described by Arunmorzhi Balajee; FFPE: formalin-fixed paraffin-embedded samples; pos.: the PCR, as well as the sequencing of the amplicon succeeded; neg.: The PCR was negative; sf: the PCR succeded, but the sequencing of the amplicon failed; n.a.: not applicable, as the corresponding PCR was not done. In yellow are indicated the samples which could be included in all phylogenetic trees (Figure 3a, 3b, 4a and 4b), in orange those which could be included in the phylogenetic analyses in the short fashion (Figure 3b and 4b).
